# A national perspective of ambulance clinicians’ perceptions, experiences and decision-making processes when assessing older adults with a head injury: a mixed-methods study

**DOI:** 10.29045/14784726.2024.12.9.3.1

**Published:** 2024-12-01

**Authors:** Jack William Barrett, Peter Eaton-Williams

**Affiliations:** South East Coast Ambulance Service NHS Foundation Trust ORCID iD: https://orcid.org/0000-0002-0040-537X; South East Coast Ambulance Service NHS Foundation Trust ORCID iD: https://orcid.org/0000-0001-5664-3329

**Keywords:** clinical decision making, head injury, older adult, paramedic, traumatic brain injury

## Abstract

**Introduction:**

UK ambulance services employ diverse models of care, resulting in 40–60% emergency department (ED) conveyance rates. Head injury conveyance rates for older adults (60 years and over) remain high (60–70%), despite most being mild. This research aimed to explore ambulance clinicians’ perceptions, experiences and decision-making processes when assessing older adults with head injuries, considering the various factors influencing their clinical decisions.

**Methods:**

This study used a mixed-methods sequential explanatory design comprising an online survey and one-to-one interviews with patient-facing ambulance clinicians in the UK. The survey, distributed through nine ambulance services and via social media, gathered data about clinicians’ experiences, confidence levels and perceptions when assessing older adults with head injuries. It focused on exposure frequency, confidence in assessing asymptomatic patients, perceived risks of medications and confidence in available decision tools. The subsequent interviews delved deeper into the survey responses.

**Results:**

A total of 385 participants were recruited, predominantly male paramedics (61%), with a median age of 35 years and a median of eight years of ambulance service experience. Participants reported frequent encounters with older adults with head injuries, and expressed high confidence in assessing visible injuries but lower confidence in conducting neurological examinations. Participants found NICE and JRCALC guidelines satisfactory, and reported confidence in conveying patients to the ED but less confidence in alternative referrals or discharges. The interviews revealed two overarching themes: guideline-based care and patient-centred care, with sub-themes emphasising the importance of shared decision making, collaboration with other healthcare professionals and safety-netting strategies.

**Conclusion:**

Although clinicians express confidence in using clinical guidelines for ED conveyances, they often find such guidance overly prescriptive and struggle to translate them for individual cases. There is a need for more patient-centred, holistic decision making, especially considering the unique aspects of head injuries in older adults. Challenges include fear of poor outcomes, limited feedback on patient outcomes and low confidence in making referral or discharge decisions. Specific guidelines tailored to this demographic, as well as improved support services, may aid in reducing unnecessary ED conveyances.

## Introduction

The UK’s healthcare services are adapting to new working models to manage the demands of an ageing population (McKee et al., 2021). Ambulance services in the UK have evolved from what was predominantly a transport service to one that now includes both ‘see and treat’ and ‘hear and treat’ models in response to the developing needs of the communities they service, the increasing demands on healthcare services and the progression of the paramedic profession (Blodgett et al., 2021). This has contributed to a reduction in the number of patients conveyed to the emergency department (ED), with rates in the UK now varying between 40% and 60% (O’Cathain et al., 2018). However, head injury conveyance rates in older adults (60 years and over) are more likely between 60% and 70% (Barrett et al., 2023; Nicholson et al., 2022).

Head injuries in older adults are predominately mild (i.e. Glasgow Coma Scale (GCS) score of 13–15), and in most cases are the result of falls from standing height (Gardner et al., 2018). Only a small proportion of these patients will suffer a traumatic brain injury (TBI), such as an intracranial bleed (Fuller et al., 2020; Mason et al., 2017), of which a tiny proportion will be eligible for neurosurgical intervention (Barrett et al., 2022, 2023). However, older adult head-injured patients with a clinically significant TBI (i.e. abbreviated injury score of 3‒5) may not present with signs and symptoms reflective of this (Kehoe et al., 2016) and, consequently, paramedic identification of TBI in head-injured older adults has been reported as poor (Hon et al., 2020; van Rein et al., 2019). Ambulance clinical guidelines developed by the Joint Royal Colleges Ambulance Liaison Committee (JRCALC) provide a list of signs and symptoms to support ambulance clinicians in conveyance decision making and to highlight key indicators for transporting patients to the ED (JRCALC, 2019). Yet the approach to older adults with head injuries is complicated by these patients’ co-morbidities, frailty and prevalent use of anticoagulant and antiplatelet medications (Gardner et al., 2018). Clinical decision making is, therefore, commonly multifactorial and complex, as highlighted by Nicholson et al. (2022), who reported that the non-conveyance of older adults with head injuries by paramedics in one regional ambulance service was influenced by factors including the availability of resources, clinician perceptions of risk versus benefit and the wishes of patients and carers. Additional factors influencing ambulance conveyance decision making in the ambulance setting have been identified as the grade of clinician attending, the use of senior decision-making support and the prevalent risk tolerance level present in the organisation (Knowles et al., 2018).

Given the disproportionate conveyance rate and broad factors influencing decision making, this study aimed to understand UK ambulance clinicians’ perceptions, experiences and decision-making processes when assessing older adults with a head injury, as well as the factors and resources they draw upon to make their clinical decisions.

## Methods

A mixed-methods sequential explanatory design was implemented to achieve the study’s aim, consisting of two phases: an online survey and remote one-to-one interviews. The survey was employed to achieve an inclusive representation of current practice in the UK, and then its participants were invited to volunteer for interviews in order to provide further context.

### Online survey design and data collection

The survey was designed to explore the frequency of ambulance clinicians’ exposure to older adults presenting with a head injury and the clinicians’ perceptions of confidence in assessing and managing this patient group, especially those patients without symptoms of head injury. These are considered a complex group of patients that can present with subtle symptoms or without displaying any symptoms reflective of a TBI (Gardner et al., 2018; Kehoe et al., 2016). Participants were also asked about the perceived risk of TBI that they associated with a limited number of clinical variables previously identified as pertinent (de Wit, Merali et al., 2020). Clinical signs and symptoms associated with a significant head injury were purposefully excluded from these questions, as the authors were particularly interested in asymptomatic presentations. Perceptions of risk associated with anticoagulant and antiplatelet medications were explored, as a small but important minority of patients taking these medications may suffer an adverse outcome (Fuller et al., 2020; Mason et al., 2017). Finally, as pre-hospital identification of clinically significant head injuries is suboptimal (Hon et al., 2020; van Rein et al., 2018) and consequently appropriate triage may also be improved (Fuller et al., 2014, 2016; Newgard et al., 2013), the authors wanted to understand participants’ confidence in the clinical decision-making tools available to them. Demographic data (age, sex, ambulance trust and clinical grade) were collected but identifiable data were only suppled by those volunteering for interview.

The survey was hosted on Online Surveys (https://www.onlinesurveys.ac.uk) and used closed-ended questions with Likert scales (Supplementary 1). The questions were developed by the authors and tested using a small sample of volunteers to ensure their suitability and readability.

### Recruitment

Following Health Research Authority and local approvals, the survey was distributed by nine UK ambulance services and on various social media platforms between 25 August 2022 and 30 September 2022. Clinicians could participate if they worked for a UK-based ambulance service in a patient-facing role. Participants were excluded if they undertook their clinical duties remotely (i.e. telephone assessment) or managed head injuries in older adults in clinical settings other than the ambulance service (e.g. primary care or ED). Participants volunteering for interviews were contacted following survey completion to arrange suitable times.

### Interview design and data collection

Interviews were conducted over Microsoft (MS) Teams or by telephone and were audio recorded and transcribed. Questions for these semi-structured interviews were pre-designed, addressing the domains included in the questionnaire. Interviews were structured to allow the participants to reflect on their perceptions of the patient population en route to an incident, their decision-making process on the scene and how they include their patients in their triaging process. These questions were further influenced by the survey responses that had been collected (Supplementary 2). Prompts were used to explore matters relevant to the study’s aims in greater depth.

### Data analysis

Frequency data were presented as counts and percentages of their total, and continuous data were presented as mean with their standard deviation (SD) or interquartile range (IQR), depending on whether the data were distributed normally or skewed. The survey data were analysed in RStudio (packages: Tidyverse, Likert and HH). Reflexive thematic analysis was employed on interview transcripts, acknowledging the subjectivity inherent in the analytical process, while aiming to prioritise the participants’ narratives using an experiential approach (Braun & Clarke, 2021). Analysis was conducted on NVivo software (V1.5.1 QSR International 2021) by PEW, a paramedic researcher with extensive experience in qualitative analysis and clinical practice in the ambulance setting. Coding was performed inductively and predominantly descriptively on each transcript, accepting narratives as an accurate representation of participants’ perceptions. Themes were generated, reviewed and defined before being developed into a framework that attempts to represent participant views on the topics addressed honestly. Data saturation and coding validation concepts are incompatible with reflexive thematic analysis (Braun & Clarke, 2021). However, the authors have sought to describe the methods and results in sufficient detail to enable readers to judge their validity and generalisability.

## Results

### Quantitative findings

Four hundred and three participants completed the online survey; three (0.7%) were excluded because they reported not working for an ambulance service, and a further 15 (3.7%) because they were in non-patient-facing roles. Males represented over half of those surveyed (n = 235, 61%), the median age was 35 years (IQR 28‒43), paramedic was the most common clinical grade (n = 174, 45%) and the median number of years working in the ambulance service was 8 (IQR 4‒14) (Table 1). 

**Table 1.** Participant demographics.

Categories	n = 385
**Age (years)**	35 (28, 43)
**Sex**	
Male	235 (61%)
Female	142 (37%)
Non-binary	1 (0.3%)
Prefer not to say	7 (1.8%)
**Ambulance Trust**	
East Midlands Ambulance Service	16 (4.2%)
East of England Ambulance Service	6 (1.6%)
London Ambulance Service	24 (6.2%)
North East Ambulance Service	10 (2.6%)
North West Ambulance Service	109 (28%)
Northern Ireland Ambulance Service	44 (11%)
Private ambulance service	6 (1.6%)
Scottish Ambulance Service	1 (0.3%)
South Central Ambulance Service	5 (1.3%)
South East Coast Ambulance Service	38 (9.9%)
South West Ambulance Service	4 (1.0%)
Welsh Ambulance Service	18 (4.7%)
West Midlands Ambulance Service	50 (13%)
Yorkshire Ambulance Service	54 (14%)
**Clinical role**	
Ambulance practitioner	8 (2.1%)
Emergency care assistant	8 (2.1%)
HART/SORT^a^ paramedic	4 (1.0%)
Newly qualified paramedic	48 (12%)
Other	20 (5.2%)
Paramedic	174 (45%)
Paramedic manager	35 (9.1%)
Specialist paramedic – critical care	10 (2.6%)
Specialist paramedic – urgent care	17 (4.4%)
Technician	61 (16%)
**Experience (years), median (IQR)**	8 (4, 14)

^a^HART: Hazardous Area Response Team; SORT: Specialist Operations Response Team.

### Exposure and confidence

**Figure 1:**
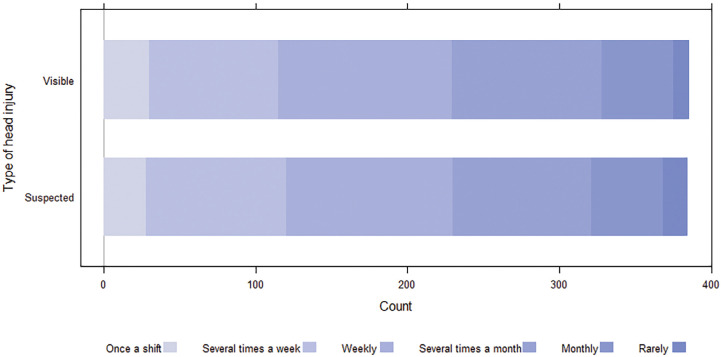
Figure 1. Participants’ self-perceived frequency of encountering visible and non-visible (suspected) head-injured older adults in their clinical practice.

**Figure 2:**
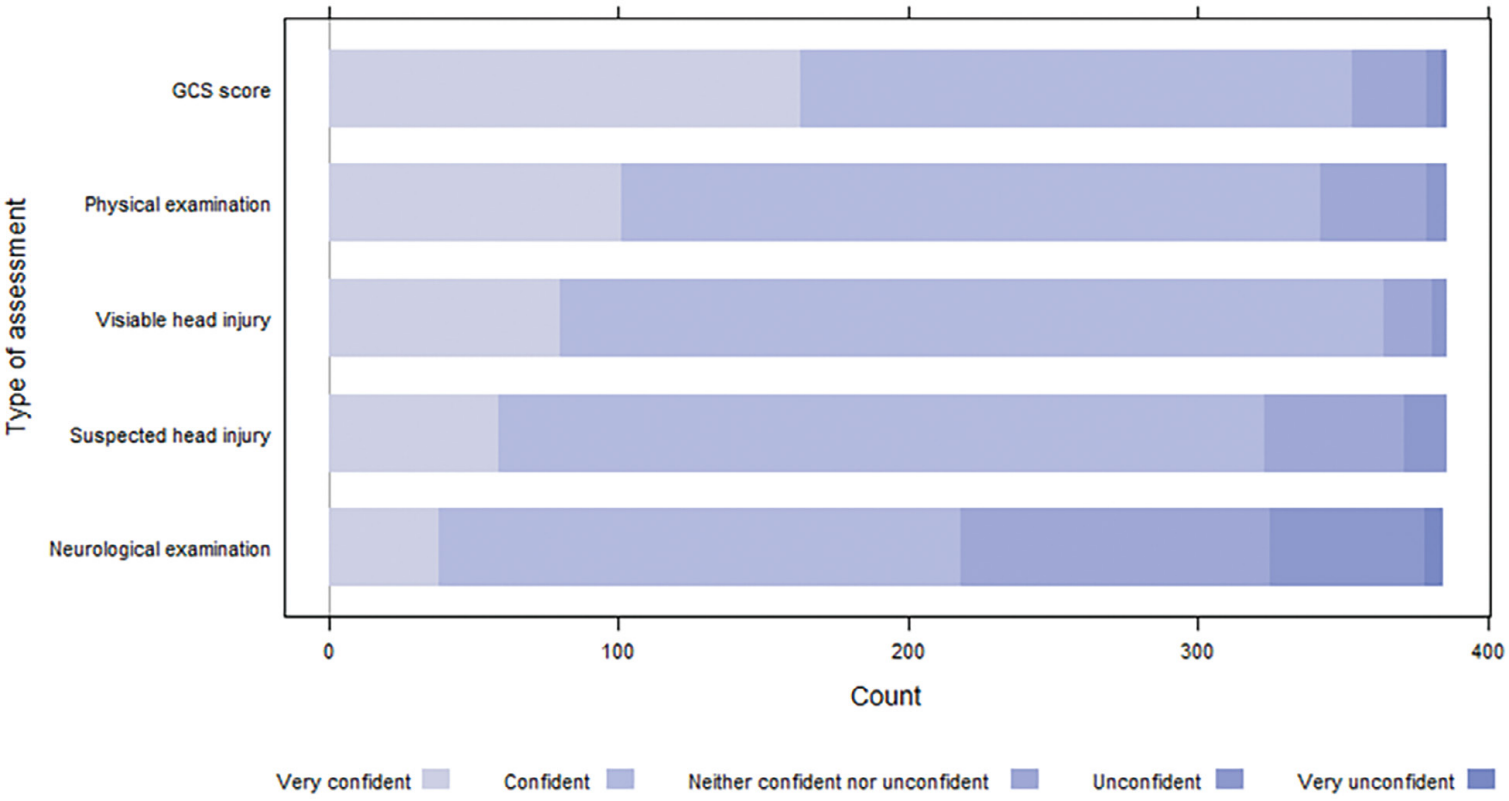
Figure 2. Participants’ self-reported confidence in assessing older adults with a head injury.

Participants reported encountering patients with head injuries (both visible and non-visible) frequently (either several times a week or weekly) (Figure 1). Figure 2 illustrates the participants’ high confidence in assessing a patient’s GCS score and performing a physical examination, and in their assessment of patients with both visible and suspected head injury (no visible injury, but had reported they hit their head). However, many participants reported being under-confident in performing a neurological examination (Figure 2).

### Clinical variables to consider in asymptomatic patients

**Figure 3:**
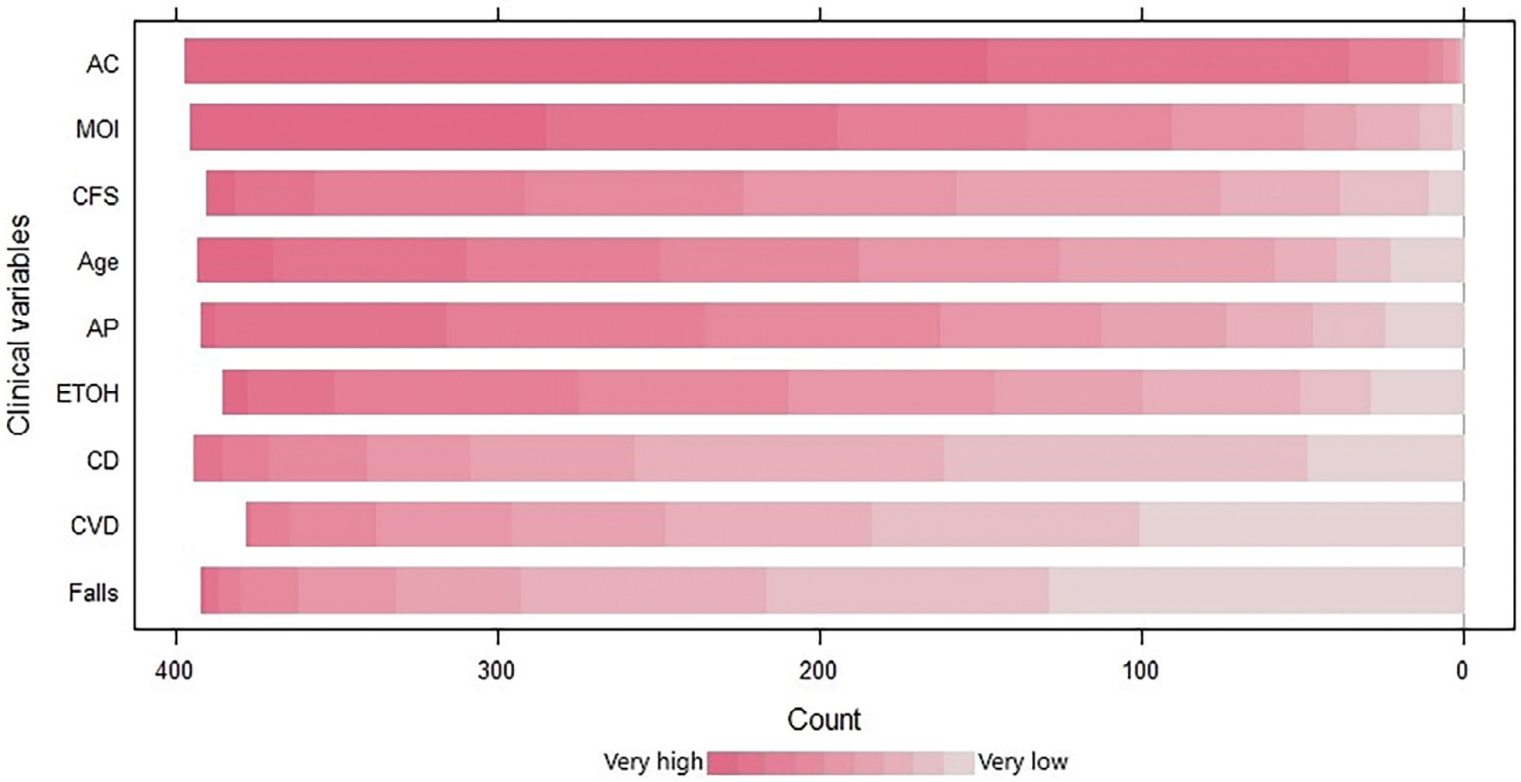
Figure 3. Participants’ perceived association of risk between clinical variables and intracranial bleeding in older asymptomatic patients.

Participants were asked to rank clinical variables and their perceived association with an intracranial bleed in a patient who did not present with head injury symptoms. Anticoagulant medication, mechanism of injury and a patient’s clinical frailty score were perceived as the highest risk factors (Figure 3).

Abbreviations – AC: anticoagulant medication; AP: antiplatelet medication; CD: cognitive decline; CFS: clinical frailty scale; CVD: cardiovascular disease; ETOH: ethyl alcohol intoxication; MOI: mechanism of injury.

### Risk of anticoagulant and antiplatelet medications

**Figure 4:**
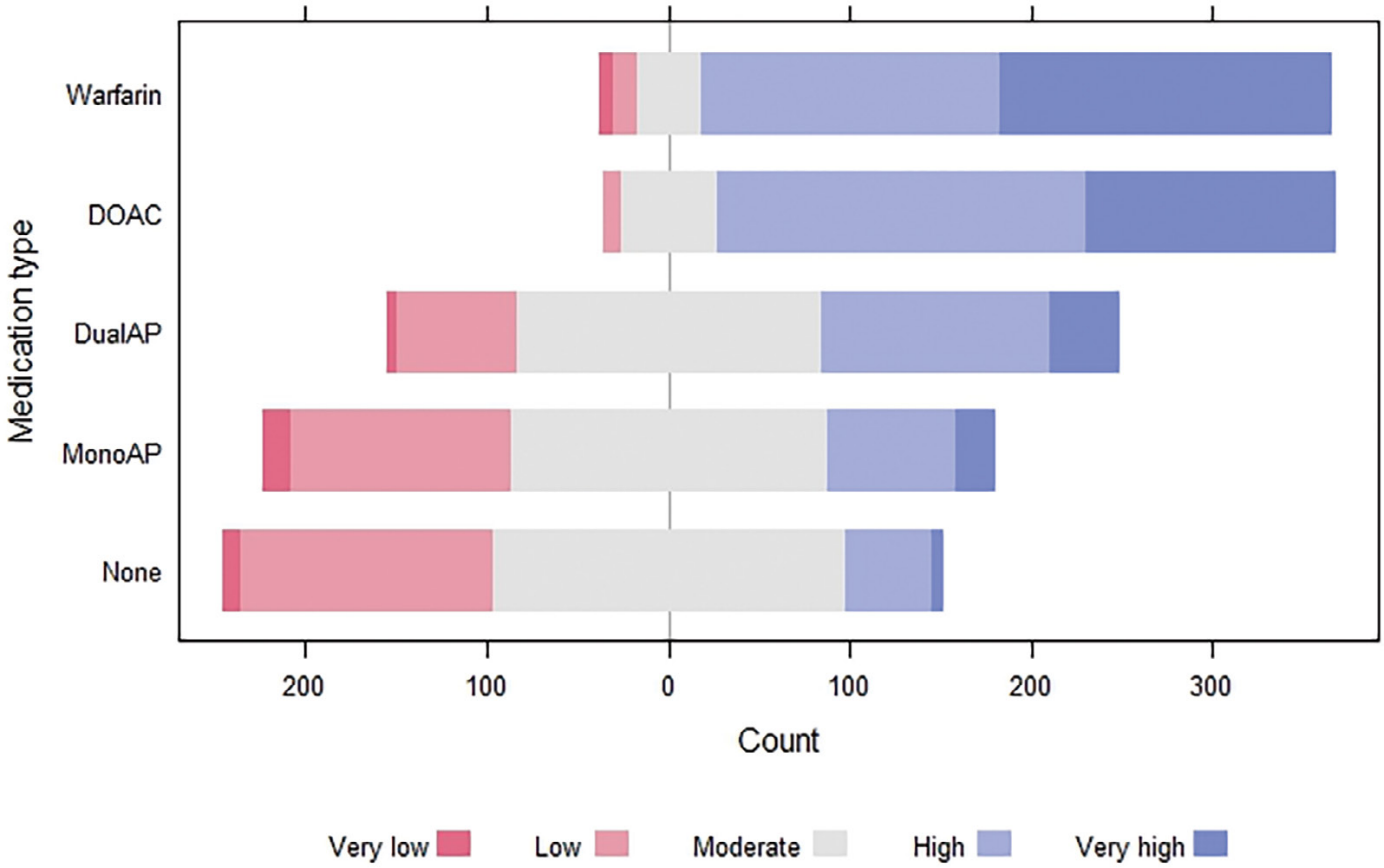
Figure 4. Participants’ perceived risk of bleeding in patients taking anticoagulant and antiplatelet medications.

In addition, when asked about the perceived risk of anticoagulant and antiplatelet medication, participants believed the risk of an intracranial bleed was high or very high in those patients taking warfarin or a direct oral anticoagulant (DOAC) (Figure 4). Although most participants felt there was a moderate risk of an intracranial bleed in patients on dual antiplatelet medications, many also felt that this applied to mono-antiplatelet therapy. A comparable proportion of participants felt that patients not prescribed either medications were also at moderate risk of an intracranial bleed.

Abbreviations – AP: antiplatelet medication; DOAC: direct oral anticoagulant medication.

### Head injury guidelines and triage decision-making support

**Figure 5:**
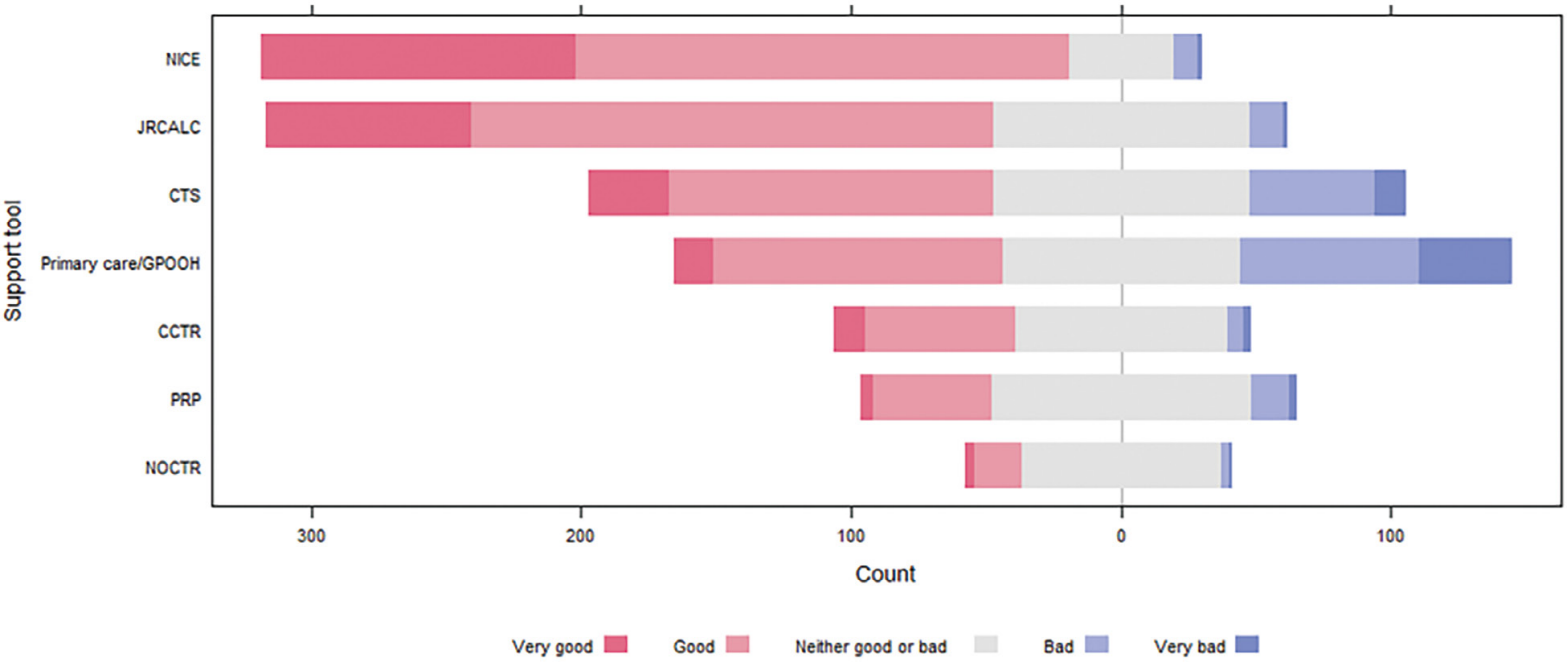
Figure 5. Participants’ perception of the quality of clinical support tools.

Overall, participants thought that head injury guidelines provided by NICE and JRCALC were good or very good (Figure 5). When used, most participants felt that clinical telephone support was also good or very good; however, participants were divided as to whether primary care or out-of-hours services were supportive in clinical decision making, and only a small proportion of participants reported that they referred directly to the Canadian Computed Tomography (CT) Head rule (Stiell et al., 2001), New Orleans CT rule (Haydel et al., 2000) or peer-reviewed papers, and they were predominately indifferent to their quality.

Abbreviations – CCTR: Canadian computed tomography rule; CTS: clinical telephone support; GP OOH: general practitioner out-of-hours; JRCALC: Joint Royal College Ambulance Liaison Committee; NICE: National Institute for Health and Care Excellence; NOCTR: New Orleans computed tomography rule; PRP: peer-reviewed papers.

**Figure 6:**
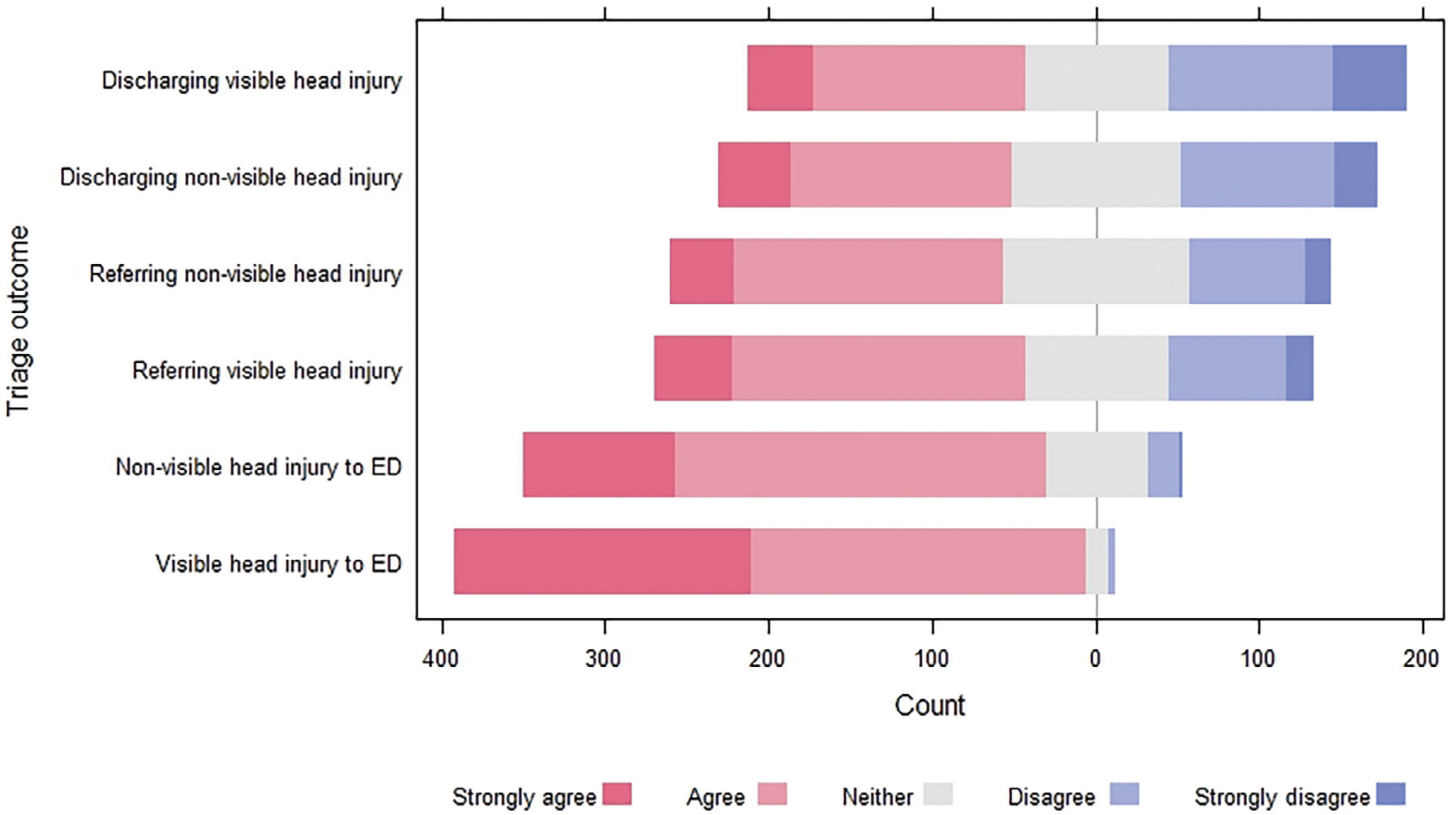
Figure 6. Participants’ perceived confidence in triaging older adults with a head injury.

When considering which patients to convey, refer or discharge, participants were very confident in their decision to convey patients (regardless of the presence of a visible head injury) to the ED (Figure 6). Less confidence was expressed for referring patients to alternative services, and even fewer participants reported confidence in discharging patients from their care.

### Qualitative findings

Twenty-one interviews were conducted between 12 September and 26 October 2022. Participants consisted of two unregistered ambulance clinicians with four years’ experience of ambulance-based clinical practice and 19 registered paramedics with a range of registered practice experience between 3.5 months and 22 years (median 6 years). Ten of the registered paramedics had previous or current experience working in specialist or advanced paramedic roles.

All participants confirmed that the presentation of an older adult with a suspected or apparent head injury was a frequent occurrence, and most indicated that during assessment the decision relating to ED transport versus management within the community was at the forefront of their minds. For some, clinical guidelines often made conveyance decisions easier. However, others reflected that without strong triggers, these patients required careful consideration of many factors to make a holistic decision on appropriate management, recognising that transporting older patients to the hospital had associated risks and benefits.

*So, everything’s about taking the holistic picture of, you know, obviously do they meet the criteria of the red criteria, do they need to go in? Well, that’s end of discussion. But, actually, if they move towards the green or the milder end of the head injury, you know, do they actually need to go in at that point or is this something that we can do within the community to keep them there safely?* (Participant 21)

Thus two overarching themes related to the clinical decision-making process were identified: clinical guidelines and a patient-centred approach. Sub-themes emerged in the patient-centred theme: the patient, the clinician and the process.

### Guideline-based care

Participants cited NICE and JRCALC guidelines as the triage tools they referred to the most. The evidence supporting these guidelines was perceived as being strong, with many participants utilising the guidance entirely prescriptively.

*I can’t really make a decision about that. It is pretty much prescribed for me. Older adults with head injuries, there is virtually no autonomy whatsoever.* (Participant 9)

Despite this, some participants were frustrated that guidance did not consider older adults as a distinct group, and some suggested that a measure of frailty would be more valuable than age in identifying risk. A few complained that whereas indications for head CT scanning were described, no information on their relative risks for the presence of injury or the likely longer-term outcomes was available to inform decision making. There was also some questioning of how ‘idealistic’ guidance translated to actual clinical practice in an NHS system under considerable pressure.

*For example, [an] anticoagulated patient should have a scan within so many hours and, especially now when we’re not going to jobs until a day later, you know, with the pressures and stuff and people are waiting 20 hours. It gives you a bit of a base that you can think, well, actually they’re already outside of being scanned and they’re all right. Do we then need to be taking these patients to the hospital?* (Participant 19)

These and other perceived grey areas within guidance led many to describe the presence of more holistic assessment and decision making.

*I think that at the end of the day, you have to use something that’s evidence based and that’s what these tools are. But I think they should be there to guide us rather than be something we have to obey slavishly. I think that any tool has its faults and when we insist that they override our own* *judgement, I think that becomes a problem.* (Participant 11)

### Patient-centred care (risks versus benefits)

#### The patient

**Mechanism and injury:** Participants highlighted the need to determine the cause of a fall and evaluate the forces that may have acted upon the head. Although all participants recognised that intracranial haemorrhage was possible without signs and symptoms in older adults, many felt that the absence of symptoms made decision making more difficult, as no clear indication to transport was apparent.

**Cognitive and physical health status:** Many participants indicated that decision making for patients in good health was easier than for those with more limited quality of life, as the risks and demands associated with ED transport and treatment were perceived as less significant. Risks associated with hospital admission for frailer patients, including hospital-acquired infection, falls, muscle wastage and cognitive decline, were highlighted. Additionally, the demands of attending ED on these patients and their carers, even if subsequently discharged, were also deemed considerable.

*You know that you’re taking this poor elderly person into a circus and they’re going to be waiting there for, you know, 8 to 10 hours.* (Participant 5)

The likely benefits for those with very poor health status were also questioned; however, this was sometimes counterbalanced by their perceived increased risk of unapparent injury.

*I have seen a huge number of patients brought in by the ambulance service who were never going to tolerate going through a scanner. The risk‒benefit is never going to be in favour of sedating those patients to put them through a scanner and, even if you do get them through the scanner, bluntly, the neurosurgeons aren’t going to be interested. Knowing they’ve got a bleed and doing something about it are two separate things.* (Participant 8, speaking of their experience working in an ED in relation to their ambulance clinical practice)

*It’s both the group that you really don’t want to take to hospital unless you have to, because it’s more likely to have bad consequences for them. But also, the group who you need to have a lower threshold to take to hospital because they’re more likely to have a bad outcome.* (Participant 7)

Significant cognitive and physical impairment was also perceived to hinder assessment. Consequently, participants described a reliance on family or carers to provide information, as well as a need to adapt assessment techniques.

. . .*especially with people with cognitive impairments. Trying to determine whether the head injury has had any significant contribution to a reduced GCS or any confusion. What is their baseline? That can certainly mix things up.* (Participant 15)

*Heel to toe and heel to shin and all of that stuff is going to be tricky for some with mobility issues. So, you probably will have to accept you’re not going to be able to do that.* (Participant 5)

**Wishes:** Participants perceived that a fear of attending hospital had become more prevalent among older adults over the past few years. Attributing this to the pandemic and to media coverage of the pressures affecting the NHS, some participants felt that certain patients consequently steered consultations purposively to avoid ED transport.

**Social situation:** Variables of dependent or independent living and the availability of family and carers affected the processes of shared decision making and safety netting that are discussed later in this article.

#### The clinician

**Confidence derived from experience:** Participants were confident in their understanding of guidance; knowledge that the guidance was evidence based and that they were working within their scope of practice gave them confidence that their management was appropriate. However, some questioned the consistency of assessment performed by ambulance clinicians, and others acknowledged the limitations of available investigations.

*But certainly, my experience is that cranial nerve assessments are not done particularly well by our more junior colleagues and paramedics.* (Participant 3)

*In the absence of a [head] CT scanner, we are very limited in the clinical examination we can do, aren’t we?* (Participant 8)

Familiarity with this presentation bred confidence, which was attributed to accumulated clinical experience. Additionally, a perception that more experienced clinicians could undertake more risk-tolerant, holistic decision making was expressed.

*I mean, when I was first qualified, everything was very risk averse, but I think probably the last two to three years I’ve got a lot more comfortable with having conversations . . . I have definitely got a lot more confident in having that discussion.* (Participant 20)

Despite this, participants conceded that they received scant feedback on the outcomes of their patients, with many citing an absence of negative feedback as a confirmation of safe practice. This limitation to practice development was also commonly acknowledged.

*For anybody who isn’t transported to hospital, then there’s no feedback . . . I mean, if you don’t get a complaint and you don’t hear anything negative, then maybe that’s the right decision, but no, it’s not that simple, is it? ... You take a patient into hospital and they don’t have a head injury, so they get sent home. It’s not necessarily the wrong decision because they’ve gone into hospital and it’s been ruled out.* (Participant 13)

**Education:** Some participants perceived that the physiological changes associated with ageing and consideration of the frail patient as a distinct group needed more emphasis in degree course content. However, a few acknowledged that their Trusts had raised awareness of issues relating to ‘silver trauma’ in recent years. A requirement for all paramedics to learn a standardised approach to head injury assessment and to be taught consultation and decision-making skills was also proposed.

**Concern for registration:** Despite common discussion of holistic decision making, many participants also expressed fear that poor patient outcomes would result in an investigation by their Trusts and that any deviance from guidelines would not ultimately be supported, endangering their professional registration and employment.

*I still would be very nervous going against NICE and I wouldn’t be supported in doing that by [my Trust] or by anyone else.* (Participant 11)

#### The process

**Shared decision making:** All participants highlighted that decision making was a process shared with the patient and, particularly in the presence of cognitive impairment, the patient’s family or carers. Despite all recognising a patient’s right to make a ‘bad’ decision, some acknowledged that they sometimes adopted a paternalistic approach to persuade patients to accept ED transport. Others claimed to be more accepting of a decision to refuse transport ‘against advice’ but stressed the need to document this comprehensively and to subsequently facilitate as much safety netting as achievable.

*If the patient is refusing against my advice to go to hospital when I’m recommending it, I’d much rather safety net that patient than do nothing.* (Participant 16)

**Collaborative decision making:** Involving other healthcare professionals in decision making was also commonly discussed; this may be a crewmate, advanced or specialist paramedics on dedicated decision-making support phonelines or a patient’s own general practitioner (GP). This was perceived as valuable, though it was generally seen as seeking confirmation or sense-checking of a decision already made.

*I want to talk through my decision making and my justification for leaving that person home and just check that that what I’m saying is sound.* (Participant 12)

**Safety netting and referral:** Patients that were not transported to hospital were told of signs and symptoms that might indicate the presence of a TBI, which should initiate a recontact. Family and carers were similarly informed and requested to check on patients regularly, which was deemed of particular importance for those with cognitive impairment. Some acknowledged the risk inherent in this safety netting.

*We can give you the safety netting for what to look out for, but it could happen very quickly. We can safety net you as much as we like but ultimately that could happen really quickly.* (Participant 17)

The presence, out-of-hours accessibility and capacity of community services to accept and respond to referrals were perceived as inconsistent. Additionally, it was considered that community care providers did not commonly accept patients with head injuries without an initial ED assessment.

*I’m a big fan of referring to community services, referring to GPs, if I don’t feel like hospital is the best place. But I think with this particular patient group, older adults with head injuries, I have tried multiple times to find an alternative and, from experience, nobody will accept them.* (Participant 13)

**Destination decision making:** Most participants indicated that they were confident to triage patients requiring direct transport to major trauma centres (MTCs) with neurosurgical capability, but viewed using their Trust’s major trauma decision tool (MTDT) and phone contact with the ‘Trauma Desk’ as integral to this process. Participants were less certain about whether identifying older adults with TBI was a pre-hospital responsibility or beyond the scope of an MTDT, particularly in determining which patients might benefit from neurosurgical care. They were unclear on how they would perform accurate identification, and while acknowledging that accessing the right hospital the first time was ideal, there were concerns that increased demands upon ambulance service and MTC resources may not necessarily result in better patient care.

*I think it’s the right thing to take them to a local trauma unit. Let them get scanned and if there is a need for a secondary transfer after that, to do it that way. Because otherwise we are just going to swamp major trauma centres with a lot of patients who don’t really benefit from that expertise.* (Participants 8)

## Discussion

Our survey of 385 UK ambulance clinicians revealed that head injuries in older adults are a frequent presentation in their clinical practice. These clinicians generally feel confident in assessing patients, regardless of a visible injury. However, our interviews revealed that, although this confidence is attributed to exposure, feedback on the associated outcomes for patients attended is rare. An absence of negative feedback is often interpreted as confirmation of practice, and this limitation to clinical development was recognised by participants and has previously been identified by ambulance clinicians many times (Eaton-Williams et al., 2020; Wilson et al., 2022). Our findings suggest that clinical decision making regarding managing older adults with head injuries is multifaceted, influenced by exposure, confidence, guideline adherence, patient-centred considerations, clinician experience and system-level factors. NICE and JRCALC guidelines are frequently used to support decision making and they are believed to be of high quality, factors that likely contribute to clinicians reporting high confidence in conveyance decisions. However, there was also a perception that guidelines are too prescriptive and do not permit consideration of all the variables identified that should influence the decision-making process. Clinical experience was one factor that facilitated adopting a more patient-centred approach, and fear of poor outcomes leading to reprisal was identified as a barrier. These findings support the work conducted by Nicholson et al. (2022), who similarly interviewed 10 paramedics in one regional UK ambulance service. Our study indicates that there remain struggles to translate clinical guidelines to individual patients possessing a myriad of pertinent considerations. This may, in part, reflect that current guidelines are derived from evidence relating to populations across the lifespan, and the causes of head injuries in younger adults, adolescents and paediatrics differ significantly from those in older adults, who predominately suffer head injuries due to falls (Gardner et al., 2018; Pozzato et al., 2019; Skaansar et al., 2020; Tiruneh et al., 2020; Yamagami et al., 2019). Therefore, clinicians may benefit from guidelines including further considerations relating specifically to older-adult head injury presentations.

This struggle to translate guidance is also likely to explain the high conveyance rates associated with this patient population (Barrett et al., 2023; Nicholson et al., 2022). However, a more patient-centred or holistic approach is increasingly advocated for older adults, as it is recognised that conveyance to the ED may not always be in their best interests, and alternative management plans may be recommended (Aminzadeh & Dalziel, 2002). Older adults experience longer waiting times and may struggle to advocate for themselves in the ED (Cetin-Sahin et al., 2020; Mwakilasa et al., 2021). Despite the absence of clinical guidelines specific to this population, our participating ambulance clinicians demonstrated that they are cognisant of the wider needs of older adults, understand their complex presentations and frailty and are balancing that against the risk‒benefit of being conveyed to the ED. In previous work conducted by the authors, we have demonstrated that head injury patients with conditions such as dementia and Alzheimer’s disease are more likely to be non-conveyed and stay at their home address (Barrett et al., 2023).

Geriatrician-led support services are becoming increasingly available to assist ambulance clinicians in their decision making and to prevent avoidable ED admissions (Jones et al., 2023). Accordingly, patient wishes, their social situation, access to decision-making support and the capacity and capability of referral pathways were all identified by our interviewees as factors influencing conveyance. However, our survey participants still indicated lower confidence in making referral or discharge decisions than in ED conveyance, indicating that further developments supporting appropriate non-conveyance may still be required.

In asymptomatic patients, survey participants considered anticoagulant medications, mechanism of injury and clinical frailty scores to be the leading factors likely to be related to a patient having an intracranial bleed following a head injury. Dual or mono antiplatelet therapies were also perceived to be a moderate risk. Interestingly, Barrett et al. (2023) demonstrated that although anticoagulant or antiplatelet medications (including monotherapy aspirin) were strong predictors of conveyance, only clopidogrel was found to be associated with an intracranial bleed on a head CT scan in their study of 3545 patients attended by a UK ambulance service for head injury. Hence, incorporating some further insight into NICE and JRCALC guidelines relating to the relative risks associated with these medications and other variables might similarly benefit a reduction in avoidable conveyances.

There are several limitations to this study, which should be acknowledged. While this is the largest survey to date that has explored clinical decision making in older adults presenting to the UK ambulance service with a head injury, it should be remembered that the views expressed here represent only a small proportion of the clinical staff nationally. Additionally, despite representation from all participating services, some were better represented than others. Both of our participant samples were achieved using a volunteer method and, as such, they may possess an inherent non-response bias (Best and Harrison, 2009). Survey design, conducting interviews and their subsequent analysis will also have introduced a degree of bias, though using mixed methods is a strength in our study (Bickman and Rog, 2009) and the views expressed are consistent with a previous regional study (Nicholson et al., 2022). Finally, it is worth noting that clinicians were asked about clinical variables that have only recently been presented in the literature (de Wit, Parpia, et al., 2020), and as such, participants may not be aware of the association of these factors with significant head trauma beyond those in clinical guidelines.

## Conclusion

Our study reveals that UK ambulance clinicians frequently encounter older adults with head injuries and that they have high confidence in using related guidelines and in their decision making to convey patients to the ED. However, many find guidelines prescriptive and challenging to translate to individuals. Clinicians feel that this patient population may benefit from a more holistic judgement of the risks and benefits of different management plans. Decision making to refer or discharge patients is done with less confidence and is influenced by multiple factors pertaining to the patient, the clinician and the support services available. Evolving current guidelines so that they may better serve this patient population, with further information on the relative risks associated with common variables and improving the capability and capacity of alternative pathways, may help safely reduce the rate of ED conveyances for older adults sustaining a head injury. 

## Author contributions

JB designed the study, JB and PEW designed the survey and the interview questions. Survey data were analysed by JB, and PEW analysed the interview data. Both authors contributed to the writing of the article. JB acts as the guarantor for this article.

## Conflict of interest

None declared.

## Ethics

Approval to conduct this study was provided by the Health Research Authority (22/HRA/2236).

## Funding

This study is funded by the College of Paramedics Small Research Grant.
